# Microemulsion: New Insights into the Ocular Drug Delivery

**DOI:** 10.1155/2013/826798

**Published:** 2013-06-27

**Authors:** Rahul Rama Hegde, Anurag Verma, Amitava Ghosh

**Affiliations:** ^1^School of Pharmaceutical Sciences, IFTM University, Lodhipur Rajput, Moradabad 244102, India; ^2^Bengal College of Pharmaceutical Sciences & Research, West Bengal, Durgapur 713 212, India

## Abstract

Delivery of drugs into eyes using conventional drug delivery systems, such as solutions, is a considerable challenge to the treatment of ocular diseases. Drug loss from the ocular surface by lachrymal fluid secretion, lachrymal fluid-eye barriers, and blood-ocular barriers are main obstacles. A number of ophthalmic drug delivery carriers have been made to improve the bioavailability and to prolong the residence time of drugs applied topically onto the eye. The potential use of microemulsions as an ocular drug delivery carrier offers several favorable pharmaceutical and biopharmaceutical properties such as their excellent thermodynamic stability, phase transition to liquid-crystal state, very low surface tension, and small droplet size, which may result in improved ocular drug retention, extended duration of action, high ocular absorption, and permeation of loaded drugs. Further, both lipophilic and hydrophilic characteristics are present in microemulsions, so that the loaded drugs can diffuse passively as well get significantly partitioned in the variable lipophilic-hydrophilic corneal barrier. This review will provide an insight into previous studies on microemulsions for ocular delivery of drugs using various nonionic surfactants, cosurfactants, and associated irritation potential on the ocular surface. The reported *in vivo* experiments have shown a delayed effect of drug incorporated in microemulsion and an increase in the corneal permeation of the drug.

## 1. Introduction

**Figure 1:**
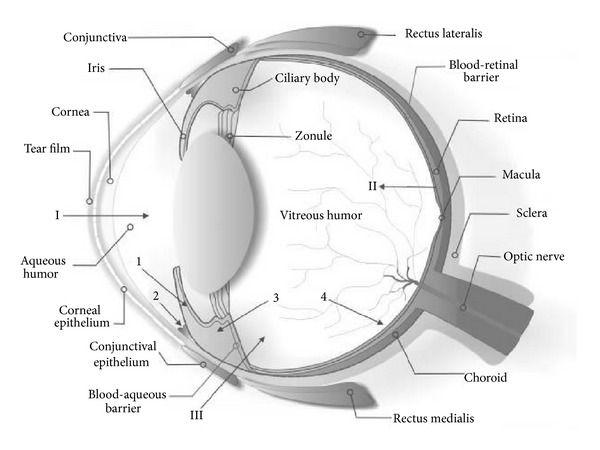
Schematic illustration of ocular structures and barriers. The primary physiologic obstacle against topically instilled drugs is the tear film. The cornea is the main route for drug transport into the anterior chamber (I). The retinal pigment epithelium and the retinal capillary endothelium are main barriers against systemically administered drugs (II). Intravitreal injection is an invasive strategy to reach the vitreous (III). Administered drugs can be carried out of the anterior chamber by venous blood flow after diffusion across the iris surface (1) or by aqueous humor outflow (2). Drugs may be removed from the vitreous cavity through diffusion into the anterior chamber (3) or by the blood-retinal barrier (4). Figure 1 is taken from Barar et al., 2009 [6].

The human eye is a complex structure designed in such a way that its anatomy, physiology, and biochemistry render it almost impervious to foreign agents, including drugs. The human eye has two segments, that is, anterior segment (cornea, conjunctiva, etc.) and posterior segment (vitreous humor, retina, etc.) as shown in detail in Figure 1. The human corneal epithelium represents one of the major rate-limiting barriers which hinders permeation of hydrophilic drugs and macromolecules. Another rate-limiting barrier is stroma which prevents diffusion of highly lipophilic drugs due to abundant hydrated collagen contents [1]. Other significant barriers include lacrimal fluid secretion and lachrymal fluid-eye barriers. Considering these barriers, it is very challenging to develop ocular drug delivery systems which can circumvent these protective barriers and deliver the drug to the posterior segment of the eye without causing permanent tissue damage [2]. Conventional dosage forms like ophthalmic solutions, suspensions, and so forth, are now primordial as they can only deliver the drug to the anterior segment of the eye but not to the posterior segment. To reduce the frequency of instillations per day, several gel formulations were developed consisting of water soluble polymers that increase the viscosity of the solution, thereby improving the residence time of drug in *cul de sac*. However, they were not much popular as they tend to blur the vision [3]. Semisolid preparations such as petrolatum-based ointments presented problems for years because they could not be filtered to eliminate particulate matter and could not be made truly sterile, and no adequate tests had been devised to indicate the suitability of added preservatives. These preparations, however, occupy a position of minor importance since they are ill-accepted on account of their greasiness, vision-blurring effects, and so forth, and are generally used as night-time medications. Novel drug-delivery systems like intraocular devices require intravitreal procedures and often suturing and, hence, can cause significant discomfort with chance of infection in the patients. Noninvasive ocular inserts also suffer from the disadvantages of foreign body sensation in the eye, membrane rupture; unnoticed expulsion from the eye [4]. *In situ* gelling systems undergo a viscosity change when administered into the eye, thereby favouring precorneal retention and when laden with nanoparticles, they can deliver the drug to the posterior segment of eye. But these systems are often difficult to develop and scale up [5]. Hence, there is a strong need to formulate ocular drug delivery systems which not only provide improved ocular bioavailability but also extended drug effect in targeted tissues. The latter requisite is very important since patients suffering from the disease of posterior segment have to take the drug throughout his/her life. These prerequisites have been appropriately reported in the literature through the use of microemulsions (MEs).

## 2. Microemulsion Science

**Figure 2:**
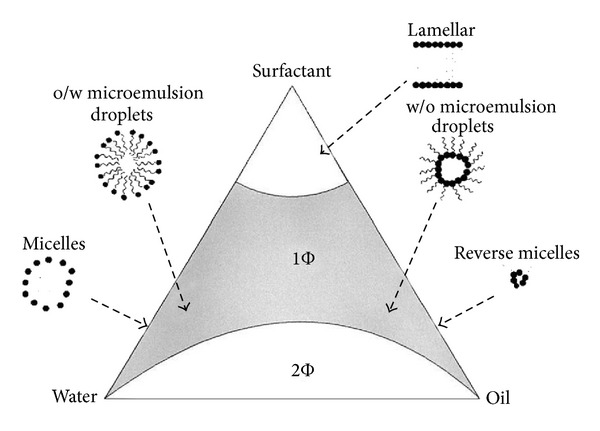
A model pseudoternary phase diagram, with the region of existence of o/w ME, w/o ME micelles, reverse micelles, and bicontinuous two-phase system with three corners representing oil, water, and surfactant. Figure 2 is taken from Lawrence and Rees, 2000 [10].

MEs are thermodynamically stable-phase transition systems, which possess low surface tension and small droplet size (5–200 nm), which may result in high drug absorption and permeation, and hence, strong possibility of drug delivery to the posterior segment of the eye. The term ME was first coined by Hoar and Schulman in 1943 [7]. Scientifically, a ME is a system of water, oil, and an amphiphile, frequently in combination with a cosurfactant, which is a single optically isotropic and thermodynamically stable liquid solution. Pharmaceutically, MEs are colloidal nanodispersions of o/w or w/o types stabilized by a surfactant film. The formation of ME along with various colloidal phases is diagrammatically explained in Figure 2. MEs are generally formed spontaneously, without any significant energy input, by mixing an oil phase with an aqueous phase containing a primary surfactant and a cosurfactant, which is usually a medium-chain-length alcohol [8]. During the mixing, the primary surfactant is adsorbed at the oil/water interface and determines the initial curvature of the dispersed phase. The required curvature for the surfactant film to attain the minimal interfacial tension is assisted by the presence of a cosurfactant. This mixed monolayer of surfactant and cosurfactant at oil/water interface can exert a two-dimensional surface pressure. The crowding of the mixed surfactants system at the interface produces stress in the system and releases it. The interface bends with the expansion of the film on one side to maintain a balance with the other side until the surface pressure at both sides of the interface becomes constant [9]. At this point, the system can be thermodynamically stable swollen mixed micelle (o/w) or inverse mixed micelle (w/o) system. The size and shape of the dispersed nanodroplets are mainly governed by the curvature free energy and are determined by the bending elastic constant and curvature of the surfactant film. The elasticity of the film depends not only on the surfactant type and the thermodynamic conditions, but also on the presence of additives like alcohols, electrolytes, block copolymers, and polyelectrolytes.

## 3. Theories of Microemulsion Formation

The three main theories of ME formation are briefly discussed here: interfacial or mixed film theory [11], solubilization theory [12], and thermodynamic theory [13]. According to the thermodynamic theory of stabilization, ME forms spontaneously because of the low value of interfacial tension on account of the diffusion of surfactant in the interfacial layer and to the major entropy contribution that depends on the mixing of one phase in the other in the form of numerous small droplets. In the mixed film theory, the interfacial film is considered to demonstrate dissimilar behavior towards the aqueous and oily segment of the interface. The solubilization theory considers ME as swollen micellar systems, in which water or oil is solubilized the reverse micelle structures to form one-phase system (Figure 2). However, despite of all the theories of ME formation, the reduction of the interfacial free energy to a very low value is of prime importance in the ME formation.

## 4. Formulation Aspect of Microemulsion

### 4.1. Selection of Lipid Phase

Many types of lipids are available, which include vegetable oils, glycerides, partial glycerides of medium-chain and unsaturated long-chain fatty acids, and polyalcohol esters of medium-chain fatty acids [14]. The physicochemical properties of the lipids must be known and understood to use them in the development of ocular MEs. Certain lipids, especially triglycerides, are completely lipophilic with HLB values of zero or close to zero because of the absence of any hydrophilic moiety. On the other hand, in case of lipids with hydrophilic moieties, there can be difference in the degree of hydrophilicity. Further, it should be also considered that most of the commercially available lipids are not pure species, rather they are mixtures of lipids with differing hydrophilic-lipophilic properties and fatty acid chain lengths. This will create difficulty when a combination of lipids with different hydrophilic-lipophilic properties is selected as lipid phase. Formation of ME with high-molecular-weight oils such as triglycerides is difficult as they contain long-chain fatty acids which are difficult to penetrate the interfacial film formed by surfactants/cosurfactant to assist in the formation of an optimal curvature [15, 16]. For this reason, smaller-molecular-weight oils (e.g., medium-chain length triglycerides are more preferred). Hydrocarbon esters of medium-chain fatty acids play an optimal role in the formation of ME and are most frequently used as an organic component of ophthalmic ME, enlisted in Table 1.

### 4.2. Selection of Surfactant(s)

The selection of surfactant system is one of the most critical steps in the design of a ME system. In ME, solubilization of oils is much greater than most micellar solutions. For one surfactant molecule, it may be possible to dissolve 10–30 oil molecules (o/w ME) or 10–300 water molecules (w/o ME). The surfactant(s) must solubilize and reduce the interfacial tension to ultra low level (<10^−3^ mN/m) between the oil and aqueous phases [9]. It is this very low interfacial tension that leads to the spontaneous emulsification of oil and water or vice versa [17]. The concentration of surfactant must be high enough (10–40%) to stabilize the nanodroplets produced by ultra low interfacial tension. Further, understanding of the partitioning behavior of surfactant in water, oil, and the interface is very important for the formulation of ME. Surfactants must exhibit appropriate phase behavior and fast equilibrium/coalescence time to minimize mass transfer and kinetic effects. In a phase behavior study, known volumes of oil and surfactant solutions in aqueous medium are placed in graduated tubes, which are then sealed. Then, the contents are mixed and allowed to equilibrate. The resulting phases of water/surfactant/oil appearing in the graduated tube and positions of their interfaces are noted. The difference in interface reading before and after equilibration is used to calculate the solubilization of oil by surfactant. All the phase behavior experiments should be conducted at surfactant concentrations greater than critical micelle concentration (CMC) of the surfactant under investigation [18]. Phase diagrams are also useful in the formulation of MEs as a means of delineating the area of existence of the ME region. These diagrams are generally constructed by plotting the observation obtained from titration of a monophasic solution of oil, surfactant(s), and cosurfactant with water. After each addition of water, the container is stoppered to minimize loss of volatile components, and the system is examined for clarity, birefringence, flow properties, and stability which is also explained later in the method of microemulsion preparation section. After the approximate determination of ME region, a more detailed study of this region of the phase diagram is required to assess long-term stability of the ME.

Other desirable characteristics for surfactants include no or very low ocular toxicity, and the ability to biodegrade neither too quickly nor too slowly. Surfactants which may be employed include both ionic agents such as cationic, anionic, or zwitterionic and nonionic agents or mixtures thereof. Among the various classes of surfactants nonionic surfactants are more versatile functional agents because of their improved solubilization characteristics: nonirritancy, ability to prolong precorneal retention with enhanced permeability. In general, all the surfactants to be used in ophthalmic ME must be subjected to extensive ocular irritation/toxicity studies because large amount of surfactant is required for the ME formation [19–21]. Nevertheless, very little research has been carried out on the toxicity of surfactants in ME form. One has to be very careful and make sure that the ocular irritation does not persist. A tabulated list of surfactants as per the available literature is provided, which can be used in the formation of ophthalmic ME in Table 2.

### 4.3. Selection of Cosurfactant

One of the major considerations in the formulation of ME is the flexibility of the interface to promote the formation of ME. For this purpose, surfactant(s) are often combined with a cosurfactant. The penetration of cosurfactant into the interfacial film produces a more fluid interface by allowing the hydrophobic tails of the surfactants to move freely at the interface. Sufficiently low fluidity and low surface viscosity of the interfacial film results in the formation of nanodroplets with small radius of curvature (50–500 Å). Generally, low-molecular-weight alcohols and glycols with chain length ranging from C_2_ to C_10_ are used as cosurfactants in preparing stable ME [16, 22]. It is reported that the chain length of alcohols is inversely proportional to the ocular irritation potential. Among various alcohols used, aliphatic *n*-alcohols with carbon chain length of 3–8 were ranked as strong irritants while ethanol was ranked as a moderate irritant. 1,2-Alkanediols with carbon chain length of 5–8, previously reported as nontoxic substitutes to *n*-alcohols, were found to be strong irritants while those with carbon chain length of 2-3 were observed to be only slightly irritating. It is also seen that ME prepared by incorporation of long carbon chain alcohols (pentanol, hexanol) as cosurfactant showed signs of ocular irritation, whereas that of short carbon chain behaved as mild irritants. Ruth and coworkers compared the efficacy of butanol and ethanol as cosurfactants in ME constituted of isopropyl myristate (IPM), egg lecithin, and water. The quantity of ethanol required for the preparation of ME is seven times higher than the quantity of butanol. The difference in efficacy between the cosurfactants is based on the length of the carbon chain [23]. The distribution of the alcohol between the interface and the continuous aqueous phase is based on its hydrophilic character. The ethanol has an interface/water distribution coefficient lower than the butanol. Therefore, its higher solubility in water requires the use of higher quantities in order to obtain an interface with similar mechanical properties to that obtained by using butanol. It has also been reported in some isolated studies that [23–25] clear stable ME can be achieved without the use of cosurfactant; the MEs so prepared were found to be practically nonirritant. A list of available cosurfactants is given in Table 3.

## 5. Charge Effect

Attempts have been made to prolong the time of residence in ocular tissues followed by topical instillation by means of electrostatic adhesion of droplets over the corneal surface. It was initially believed and now has become clearer from many reports in the literature that an occurrence of electrostatic attraction between the cationic emulsified droplets and anionic cellular moieties of the ocular tissues exists [26]. As the corneal area is negatively charged, the positively charged droplets might bind to the sites. The charge is provided by a positively charged lipid, for example, stearylamine or cationic polysaccharide, for example, chitosan.

Beilin and coworkers reported that the presence of positive charge on the surface of internal phase could influence drug absorption through corneal penetration [27]. The supposition was based on the presence of negative charge on the corneal surface which would facilitate binding of positively charged droplets of the submicron emulsion [28]. Calvo and coworkers studied comparative behavior of drug release through colloidal systems, namely, nanocapsules, nanoparticles, and submicrons emulsion, the findings of which showed an increased corneal permeation of indomethacin due to incorporation of the drug in colloidal carriers instead of the electrostatic attraction between the negatively charged cornea and positively charged drug carrier system [29]. They found that the incorporation of the drug into a colloidal system facilitates the uptake of nanoglobules by the corneal epithelium without causing any damage to the cell membrane [30]. 

## 6. Methods of Preparation

There are two methods of preparing microemulsion, via phase inversion temperature and phase titration methods. However, no study has been reported yet on microemulsion for ocular delivery prepared using phase inversion technique; thus, this technique is not discussed in the current review.

### 6.1. Phase Titration Method

Phase titration is low-energy emulsification method. This utilizes the spontaneous diffusion of surfactant or solvent molecules into the continuous phase due to ultra low interfacial tension. Preparation of ME involves investigation of area of formation of single-phase region in the phase diagram which is composed of 4 corners, each of oil, water, surfactant, and cosurfactant, respectively. In this method, all the components of formulation are mixed in proportions varying from 0 to 100% representing in the phase diagram, in anticipation to obtain a clear phase. Subsequently, optimization is appropriately done based on the most clear region in the phase diagram and then to finalize the composition of most stable ME [18, 22].

## 7. Characterization of Microemulsion

ME characterization can be divided into 3 major areas, physical evaluation, electrochemical evaluation, and microscopic evaluation. Appearance, viscosity, and optical clarity provide useful information about the physical nature of the microemulsion. Osmolality is essential for physiological acceptance of the formulation by ocular tissues and is measured using osmometer whereas the surface tension essential to ensure uniform spreading on corneal surface which is determined by use of tensiometer. The presence of cubic, rod-shaped, and elongated cylindrical micelles and the transition between ME structures can be interpreted by changes in viscosity. The rheological properties of ME have been extensively reviewed by Hellweg [31], Strey [32], and Ktistis [33]. Conductivity measurements can be used to determine whether a ME is oil-continuous or water-continuous and may also be used to monitor percolation or phase inversion phenomena. Dielectric measurements have been used to investigate both the structural and dynamic features of ME. The optical clarity of ME is due to the small droplet size and is evaluated by using microscopic methods and light scattering methods which gives satisfactory results. However, the various structures arising due to internal transition in the ME demand special measurement techniques [34]. A variety of methods [35], such as freeze fracture electron microscopy, and a range of light scattering methods, such as small-angle X-ray scattering, small-angle neutron scattering, total intensity light scattering, and photon correlation spectroscopy, may be used to determine the particle size of a ME. SANS and SAXS are useful methods to determine the ME microstructure and droplet size and shape. Pulsed gradient spin echo- (PGSE-) NMR technique can be utilized in the determination of the self-diffusion coefficient of the different components of the ME. DSC is utilized in determination of the state of water in ME by distinguishing between bulk water and bound water.

## 8. Mechanism of Drug Release from Microemulsion

ME droplets exist in micelle form and various structures: droplets of oil or water, ordered or lamellar structures. The drug loaded in the ME exists mostly in the internal phase. However, at the equilibrium state, the drug can be distributed among dispersed phase, continuous phase, and surfactant interphases. The drug release from the ME can be explained by using two models. One model explains the drug diffusion throughout the droplet as rate-limiting step of drug release, whereas the other model considers the interfacial barrier between the droplet and surrounding as rate-determining step of drug release. The most acceptable model of drug release from ME described the combination of mass balance and linear dependence of mass fluxes on concentrations.

Generally, the drug release from the ME is studied by determining the mass transfer constants of the drugs through a biological membrane separating the ME from the receiver phase. The mass transfer constant is directly related to the partition coefficient of the drug in oil-surfactant-water mixtures. Release of drug from ME mainly depends on oil-aqueous phase ratio, droplet size, and distribution of drug in the phases of ME. The release pattern is further governed by the rate of transfer of drug from disperse phase to continuous phase and thereby from continuous phase through the biological membrane. It is anticipated that the permeation of hydrophilic drug through the biological membrane contacting the ME will depend on the concentration of drug in the aqueous phase of ME and vice versa in case of lipophililc drug.

## 9. *In Vitro* Models for Drug Release Kinetics from Microemulsion

Although the available diffusion models for *in vitro* diffusion kinetics may not give the exact scene happening *in vivo*. *In vivo* sink conditions and continuous clearance of the released drug by tear from surface of *cul de sac* as well of ocular tissues are difficult to maintain. The artificial cellulose membrane cannot mimic the barriers of corneal membrane. The constant volume of diffusion cell will not be able to eliminate the drug released by tear fluid turnover. So the method of diffusion cell is not representative of the real situation *in vivo*.

In previous studies, the uptake of drugs across the cornea *in vitro* has been investigated using corneal perfusion chambers [36, 37] maintaining constant volume of buffer in donor side and the receiver side. Corneal permeability was expressed as the apparent permeability coefficient

(1) $$\begin{array}{c} P_{\text{app}}=\frac{dq}{dt}\frac{1}{C_{o}A}, \end{array}$$

where *C*_*o*_ is the initial donor side drug concentration and *A* is the corneal surface area [38]. The value of *P*_app_ (cm/s) obtained describe how well the compounds penetrated the cornea from the buffer used [39]. Nevertheless, this parameter was difficult to relate with *in vivo* bioavailability as it did not take physiological and formulation variables into account. In the *in vitro* experiments, the drug remains in intimate contact with the isolated cornea; certain drugs tend to swell cornea due to intrinsic corneal toxicity. Some poor penetrants require very long incubation times to reach steady state, thereby prolonging the corneal exposure time during the experiment as a result of which it becomes difficult to maintain the corneal integrity throughout the study. Another limitation is ocular tear flow dynamics which are differed from the design of *in vitro* chamber. When a drug is applied to the eye *in vivo*, it is washed away with continuous tear flow and with the overfilling of *cul de sac*. The *in vivo* tear volume is 7–12 *μ*L with approx. 7% turnover per minute [40] which is difficult to maintain in the laboratory *in vitro* conditions.

Recently, some authors [41] studied transcorneal diffusion of the drug by using novel modified Franz diffusion chamber with a mechanism to control tear fluid turnover. They performed diffusion of timolol maleate incorporated in *in situ* gel and aqueous solution. The *in vitro* assembly consisted of four cells, each having upper and lower chambers. The upper chamber served as a donor compartment in which 100 *μ*L of drug solution/formulation was placed. The upper and lower chambers were separated by excising goat cornea. The lower chamber served as a receiver compartment that was infused continuously with simulated tear fluid at the rate of 20 *μ*L/min with the help of peristaltic pump. The whole system was maintained at 37 ± 0.5°C.

## 10. Developed Microemulsions for Ocular Therapeutics

**Figure 3:**
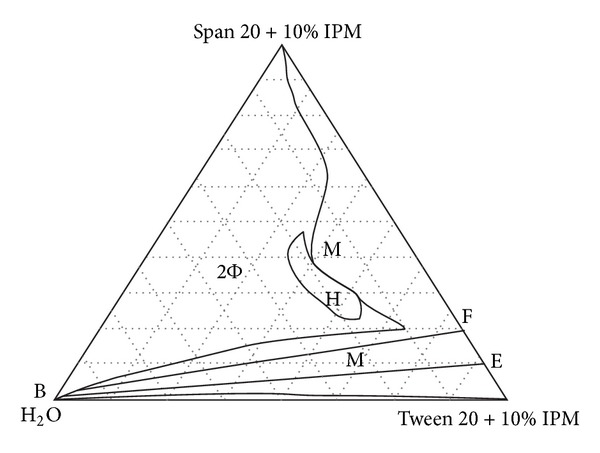
The pseudoternary phase diagram of chloramphenicol (Free) microemulsion composed of Span 20 + Tween 20 + isopropyl myristate + water showing formation of single-phase microemulsion region (M) and biphasic region (2Φ).  Figure 3 is taken from Lv et al., 2005 [46].

**Figure 4:**
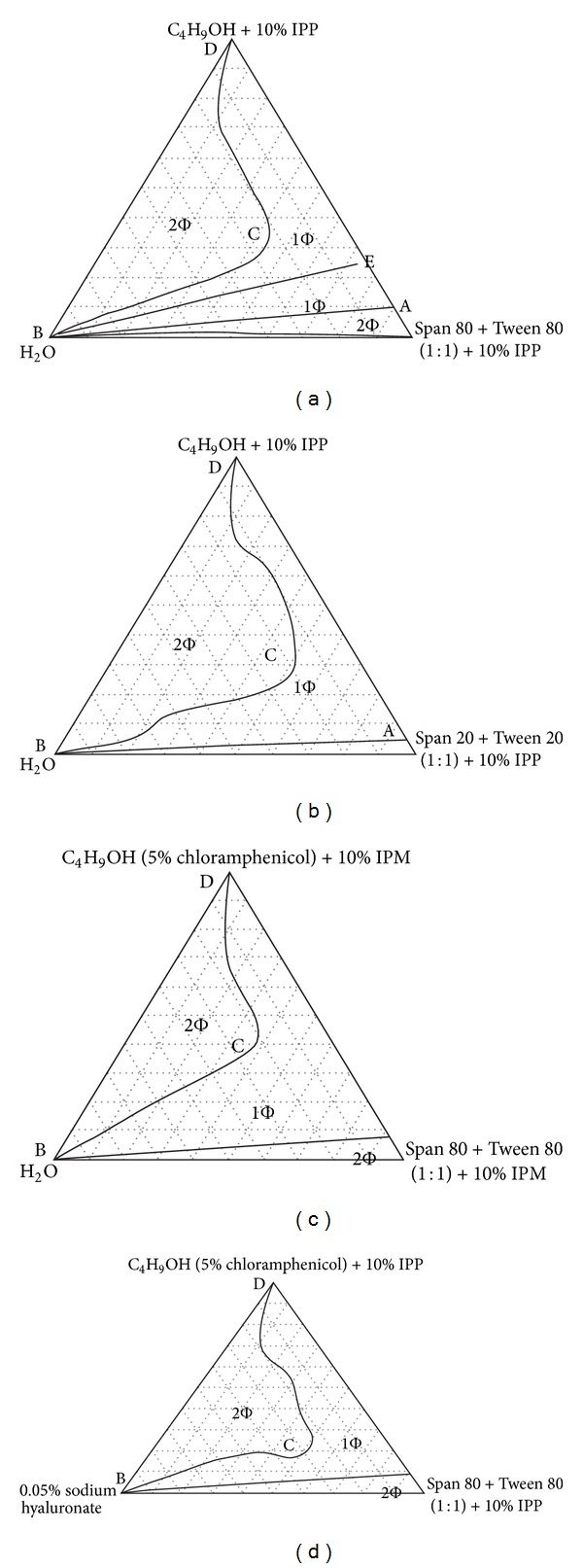
The pseudoternary phase diagrams of various systems at 25°C: (a) Span 80 + Tween 80 (1 : 1) + *n*-butanol + IPP +H_2_O; (b) Span 20 + Tween 20 (1 : 1) + *n*-butanol + IPP + H_2_O; (c) Span 80 + Tween 80 (1 : 1) + *n*-butanol (5% chloramphenicol) + IPM + H_2_O; (d) Span 80 + Tween 80 (1 : 1) + *n*-butanol (5% chloramphenicol) + IPP + 0.05% sodium hyaluronate. All the ratios mentioned above are weight ratios except the ratios of Span/Tween are molar ratios.  Figure 4 is taken from Lv et al., 2006 [25].

**Figure 5:**
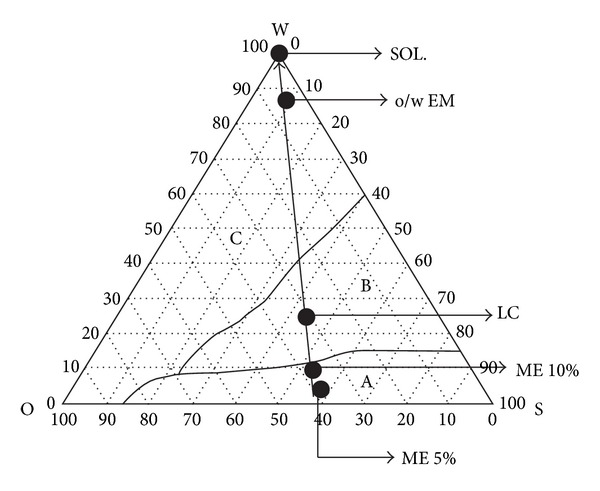
Crillet 4 system. W: 100% water; O: 100% Crodamol EO; S: 100% surfactant blend of Crill 1 and Crillet 4 (ratio of 2 : 3). (A) Systems forming water-in-oil microemulsions; (B) systems containing liquid crystals; (C) systems forming coarse emulsions. ME 5%: water-in-oil microemulsion containing 5% (w/w) aqueous phase; ME 10%: water-in-oil microemulsion containing 10% (w/w) aqueous phase; LC: lamellar liquid crystalline systems; EM: oil-in-water coarse emulsion systems; SOL: aqueous solution.  Figure 5 is taken from Chan et al., 2007 [47].

For ophthalmic applications, properly formulated MEs are reported to provide ease of application just like eye drop solutions, with added advantages of being patient friendly, due to less frequent application, improved retention, and extended drug action. Many researchers attempted successful development of ME for ophthalmic delivery of drugs as enlisted in Table 4 and the physicochemical parameters are enlisted in Table 5. The ternary phase diagrams of the above reported MEs are enlisted in Figures 3, 4, and 5.

## 11. Recent Patents on Microemulsion for Ocular Therapeutics

Many inventors filed patents on ocular drug delivery utilizing microemulsion as a delivery system; however, in this review only the patents filed in the past five years are listed in Table 6.

## 12. Conclusion and Future Prospects

ME holds significant promise for topical ophthalmic application due to their eye-drop-like consistency, nano droplet size range, and phase transition behavior. MEs may constitute an effective system for the delivery of both water soluble and insoluble drugs to the ocular tissues without compromising the convenience to the patients as well as ophthalmologists for adjustment of dose and dosing frequency according to the disease therapy. Due to the phase transition behavior, ME can form *in situ *precorneal depots resulting in improved retention and, thus, prolonged release of incorporated drug. From the researched literature, it has been found that judiciously selected lipid phase (generally medium-chain triglycerides), surfactant phase (especially nonionic), and cosurfactant can be combined with drugs in such a way that drug is released into the eye in a precise and controlled manner. The ME systems for ocular delivery have been reported to possess excellent physicochemical properties and stability. Apart from this, they are easy to fabricate and characterize. Their process of preparation is simple and inexpensive leading to easy scale-up and reduced final cost of dosage form. As has been discussed in this review, there is ample evidence that MEs may constitute efficient future ocular drug delivery systems that have the ability to penetrate different ocular tissues by circumventing the anatomical and physiological barriers, thereby completely replacing rather primitive conventional ocular drug delivery systems like eye drop, eye ointments, and so forth. MEs are expected to deliver any drug to both the anterior and posterior segments of the eye, at the right time in a safe and reproducible manner at required level. However, a wider area of further studies, such as validation of drug release for ophthalmic application together with development of new technologies, holds the future of clinical significance of MEs in the effective treatment of ocular diseases.

## Figures and Tables

**Table 1:** List of lipid phase.

Esters of fatty acids	Ethyl oleate, isopropyl myristate, and isopropyl palmitate
Monounsaturated fatty acids	Oleic acid
Saturated fatty acids/low-molecular-weight triglycerides	Capric-caprylic triglyceride (miglyol 80), octanoic acid

**Table 2:** List of surfactants commonly used in ophthalmic microemulsion.

General class	Examples
Lecithin and lecithin derivatives	Pure phospholipids (e.g., soya phosphatidyl choline) and mixed phospholipids, sodium cholate Hydroxylated phospholipids/lecithin

Glycerol fatty acid esters	Polyglycerol fatty acid esters Polyglycerol polyricinoleate Propylene glycol fatty acid esters (e.g., polyoxyethyleneglycerol triricinoleate, cremophor EL (macrogol-1500-glyceroltriricinoleate) monobutyl glycerol)

Sorbitan fatty acid esters	Span 20 (sorbitan monolaurate) Span 80 (sorbitan monooleate)

Polyoxyethylene sorbitan fatty acid esters	Tween 20 (polyethylene glycol sorbitan monolaurate) Tween 80 (polyethylene glycol sorbitan monooleate)

Others (potential cosurfactants)	Propylene glycol PEG 200

**Table 3:** List of cosurfactants.

Alkanol	Ethanol, propanol, and 1-butanol
Alkane-diols	1,2-Propane diol, 1,2-butane diol
Alkane-polyols	Glycerol, glucitol, and polyethylene glycol

**Table 4:** Brief summary of reported work on formulation development on ocular microemulsion.

Researchers	Drugs used	Surfactants	Co-surfactants	Other ingredients	Description and outcome of the study
Gallarate et al., 1988; Gasco et al., 1989 [42, 43]	Timolol	Lecithin	1-Butanol	Isopropyl myristate, octanoic acid (OA), and distilled water	The topical administration of timolol as an ion-pair with octanoate was achieved by the use of an oil-in-water ME. The areas under the curve for timolol in aqueous humour after administration of the ME and the ion-pair solution were 3.5 and 4.2 times higher, respectively, than that observed after the administration of timolol alone

Gallarate et al., 1993 [44]	Levobunolol (LB)	Lecithin	1-Butanol	Isopropyl myristate, octanoic acid (OA), and distilled water	Aqueous and aqueous-PEG 200 solutions and o/w ME containing LB coupled to OA as lipophilic ion-pair were prepared and investigated *in vitro*, in view of possible ophthalmic applications. Permeation studies in aqueous and in aqueous-PEG-200 solutions through the artificial membrane indicated a higher apparent lipophilicity of LB-OA with respect to the drug alone. The ME, which was isotonic and nonirritating to rabbit eyes, appears as a potentially interesting ophthalmic vehicle for LB

Haße and Kiepert, 1997 [45]	Pilocarpine nitrate	Macrogol-1500-glyceroltriricinoleate and lecithin	PEG 200, Propylene glycol,	Isopropyl myristate, distilled water	The authors developed o/w ME for ocular application of pilocarpine. Prolonged *in vitro * drug release was observed from ME. The miotic activity was measured on albino rabbits. For ophthalmological use, the miotic retarding effect of pilocarpine in ME turns out to be advantageous

Fialho and da Silva-Cunha, 2004 [24]	Dexamethasone	Cremophore EL	Propylene glycol	Isopropyl myristate, benzalkonium chloride, and distilled water	Developed MEs showed acceptable physicochemical properties and stability. The ocular irritation test suggested that the MEs did not provide any significant alteration to the eyelids, conjunctiva, cornea, and iris. This formulation showed greater penetration of dexamethasone in the anterior segment of the eye and also release of the drug for a longer time when compared with a conventional preparation. The area under the curve obtained for the ME system was more than twofold higher than that of the conventional preparation

Alany et al., 2006 [23]	Pilocarpine hydrochloride	Sorbitan laurate, polysorbate 80	Alkanol or alkandiol	Ethyl oleate, water	w/o MEs capable of undergoing a phase-transition to lamellar liquid crystals or bicontinuous MEs upon aqueous dilution were formulated. Results showed only formulations having cosurfactants; all other ingredients were nonirritant to rabbit eyes. It was observed that cosurfactant irritation was dependent on its carbon chain length. Precorneal clearance studies revealed that the retention of colloidal and coarse dispersed systems was significantly greater than an aqueous solution with no significant difference between MEs

Lv et al., 2006; 2005 [25, 46]	Chloramphenicol	Tween 20	Span 20	Isopropyl myristate, distilled water	Chloramphenicol was trapped into oil core or palisade layer of the o/w ME free of alcohols. Its stability was investigated by the high-performance liquid chromatography (HPLC) assays and H^1^-NMR in the accelerated experiments of 3 months. The stability of the chloramphenicol in the ME formulations was increased remarkably; the pseudoternary diagram of the ME is given in Figures 3 and 4

Chan et al., 2007 [47]	Pilocarpine hydrochloride	Polyoxyethylene sorbitan monooleate	Sorbitan monolaurate	Ethyl oleate, water	ME-based phase transition systems were evaluated for ocular delivery of pilocarpine hydrochloride (model hydrophilic drug). These systems undergo phase change from ME to liquid crystalline (LC) and to coarse emulsion (EM) with a change in viscosity depending on water content (Figure 5). The miotic response and duration of action were greatest in case of ME and LC formulations indicating high ocular bioavailability (Figure 5)

Baspinar et al., 2008 [48]	Everolimus	Poloxamer 184	Propylene glycol	Triacetin, deionized and sterile water	In this study, ocular MEs bearing everolimus were prepared for preventing corneal-graft rejection. The permeation rate of the model drug everolimus through a freshly isolated pig cornea was determined *ex vivo*. Authors concluded that prepared ME is a promising ocular formulation for preventing corneal-graft rejection

Kesavan et al., 2013 [26]	Dexamethasone	Tween 80	Propylene glycol	Isopropyl myristate, chitosan, and distilled water	The mucoadhesive chitosan-coated cationic MEs were prepared for treatment in condition of chronic uveitis. The average globule size was less than 200 nm with a positive surface charge. The developed microemulsion revealed stability for 3 months. The *in vivo * studies evidenced marked improved therapeutic effect of the incorporated steroid

**Table 5:** List of studied physicochemical parameters of various reported ocular MEs.

Authors	Drug	Physicochemical properties (mean ± SD)				
		pH	Average diameter (nm)	Refractive index	Surface tension (mN/m)	Viscosity (mPa·s)
Gasco et al., 1989 [43]	Timolol maleate	—	15	—	—	24.8 ± 0.7
Haße and Keipert, 1997 [45]	Pilocarpine nitrate	5.5–6.0	25–45	1.37–1.38	31-32	7.0–9.0
Fialho and da Silva-Cunha, 2004 [24]	Dexamethasone	6.99 ± 0.02	50.85 ± 1.24 (*n* = 6)	1.38 ± 0.01	27.79 ± 0.01	40.27 ± 0.98
Lv et al., 2006 [25]	Chloramphenicol	—	53–59.5	—	—	—
Chan et al., 2007 [47]	Pilocarpine HCl	—	<100	—	34 ± 1.6	167

**Table 6:** Recent patents filed dealing with ocular MEs.

Recent patents	Drugs used	Surfactants	Co-surfactants	Other ingredients	Description and outcome of the study
Sergio et al., WO 154985A1, 2011 [49]	Steroids (difluprednate), prostaglandin (latanoprost) NSAID ( diclofenac), antioxidant, and pegaptanib	d-*α*-tocopheryl PEG 1000 succinate	Glycerol	Vitamin E, MCT, and disodium phosphate	The inventors developed o/w ME for encapsulation of water insoluble drugs for topical ophthalmic application. The developed ME carrier remained stable for a period of 6 months displaying a particle size of 15 nm without any signs of instability or separation

Gobel, European patent EP- 2485714A1, 2012 [50]	Tacrolimus	Lecithin, decyl glucoside, span 80 (sorbitan monooleate), and brij 30 (polyoxyethylene(4)lauryl ether)	Pentylene glycol, propylene glycol, and PEG-20	Dibutyl adipate, isopropyl myristate, and tartaric acid	The transparent o/w ME for delivery of immunosuppressant agent tacrolimus is subjected to HET-CAM test and claimed to be free from signs of irritation. The particle size range varied from 5 to 100 nm. Additionally, the tacrolimus ME was found to penetrate efficiently the stratum corneum tissue and reach the dermis due to presence of lymphocyte, which is the target for the active ingredient

Carli et al., US Patent US 8414904B2, 2013 [51]	Prostaglandin analogue (latanoprost, travoprost, and bimatoprost)	Tween 80, brij 52, 56, 58	Tween 20	Ethyl oleate, miglyol 812, ricinus oil sorbitol, glycerol, chlorobutanol, and buffer (pH 7.4)	o/w MEs composed of prostaglandin formulated with two nonionic surfactants and one oily component displayed a particle size not more than 700 nm and a low zeta potential of +2 to −2 due to the use of nonionic surfactants as emulsifying agents. The formulation was claimed to be free from any signs of irritation on rabbit eyes. The ME remained stable for a period of 12 months
